# Diarrhœa

**Published:** 1857-07

**Authors:** 


### DIARRHŒA.

IT may be well for persons travelling during the summer to know, that, in case a physician is not at hand, a safe remedy, of considerable efficacy, is found in stirring a little wheat flour in a glass of cold water, until it is of the consistency of thick cream, drink it down, and repeat it several times in the course of the day, if needed. Meanwhile, eat nothing, drink nothing, and lie down, if practicable. The flour may act mechanically, not medicinally, by plugging up the relaxed mouths through which the watery particles are poured into the intestinal canal.

Here, diarrhœas are often the result of the greater coolness of morning and evening over mid-day, and the injurious effects of bad air on an empty stomach; hence, one of the most important rules for travellers, in all seasons, climes, and countries, is, NEVER FAIL TO BREAKFAST BEFORE YOU RIDE.

